# Studies on the Induction of Antigen-Specific Antibody in Anti-CD40 Cultured Human B Lymphocytes

**DOI:** 10.1155/1998/35259

**Published:** 1998

**Authors:** B. M. Schilizzi, M. C. Harmsen, T. H. The, L. De Leij

**Affiliations:** Department of Clinical Immunology University Hospital Groningen 9713 EZ The Netherlands

**Keywords:** Specific antibody, anti-CD40, human B lymphocytes, cytomegalovirus (CMV)

## Abstract

Costimulatory signals provided by T cells are required for B cells to produce specific antibody to T-dependent antigen. We have investigated the suitability of using the CD40 culture system for the proliferation and differentiation of Ag-specific human B cells using cytomegalovirus (CMV) or tetanus toxoid (TT) as antigen. We modified the CD40 culture system (CD32- transfected L cells, anti-CD40, and IL-4) by applying a sequential cytokine stimulation and compared total B-cell cultures with antigen-specific B cells preselected by panning. The detection of specific antibody became possible when antigen-selected B cells were cultured for 7 days in the CD40 system to induce clonal expansion, followed by the addition of IL-2 and IL10 for an additional 7 days to induce plasma-cell differentiation. We conclude that our intial inability to detect specific antibody in the CD40 system is due to overgrowth of nonspecific B cell clones and that selection of antigen-specific B cells by panning overcomes this problem. Induction of antigen-specific antibody production was found to be optimal when the initial contact with antigen during panning was limited to between 1 to 24 hours.


*Developmental Immunology*, 1998, Vol. 6, pp. 261–271
Reprints available directly from the publisher
Photocopying permitted by license only

© 1998 OPA (Overseas Publishers Association)
N.V. Published by license under the
Harwood Academic Publishers imprint,
part of the Gordon and Breach Publishing Group
Printed in Malaysia

### Studies on the Induction of Antigen-Specific Antibody in Anti-CD40 Cultured Human B Lymphocytes

B.M. SCHILIZZI, M.C. HARMSEN, T.H. THE and L. DE LEIJ*

*Department of Clinical Immunology, University Hospital, 9713 EZ Groningen, The Netherlands*

*(Received 16 August 1996; In final form 2 May 1997; Accepted 12 August 1997)*

Costimulatory signals provided by T cells are required for B cells to produce specific antibody to T-dependent antigen. We have investigated the suitability of using the CD40 culture system for the proliferation and differentiation of Ag-specific human B cells using cytomegalovirus (CMV) or tetanus toxoid (TT) as antigen. We modified the CD40 culture system (CD32-transfected L cells, anti-CD40, and IL-4) by applying a sequential cytokine stimulation and compared total B-cell cultures with antigen-specific B cells preselected by panning. The detection of specific antibody became possible when antigen-selected B cells were cultured for 7 days in the CD40 system to induce clonal expansion, followed by the addition of IL-2 and IL-10 for an additional 7 days to induce plasma-cell differentiation. We conclude that our intial inability to detect specific antibody in the CD40 system is due to overgrowth of nonspecific B-cell clones and that selection of antigen-specific B cells by panning overcomes this problem. Induction of antigen-specific antibody production was found to be optimal when the initial contact with antigen during panning was limited to between 1 to 24 hours.

*Keywords:* Specific antibody, anti-CD40, human B lymphocytes, cytomegalovirus (CMV)

### INTRODUCTION

Antibody responses to thymus-dependent antigens are mediated by cytokines and B-cell-surface receptor-ligand interactions provided by T helper cells. One of the important T-cell surface ligands is CD40L that interacts with CD40 on B cells and that is transiently expressed on activated T cells during the immune response (Banchereau et al., 1991; Hollenbaugh et al., 1992). Ligation of CD40 on B cells has a wide range of effects, that is, together with IL-4, it gives a costimulatory signal for proliferation (Clark et al., 1988; Banchereau et al., 1991; Rousset et al., 1995) and heavy-chain switching (Gascan et al., 1991), whereas such ligation together with IL-2, IL-10, or IL-15 induces antibody secretion (Rousset et al., 1992, 1995; Armitage et al., 1993). Furthermore, CD40 and B-cell antigen receptor (BCR) dual triggering of resting B lymphocytes turns on a partial germinal center phenotype (Galibert et al., 1996A), and CD40 ligation is thought to be essential in the rescue of germinal center B cells from apoptosis after

*Corresponding author.

somatic mutation in the germinal center (Liu et al., 1992). *In vitro*, the continued stimulation of germinal center B cells by anti-CD40 in the presence of IL-2 and IL-10 promotes the generation of $CD38^{-}CD20^{+}$ memory B cells, whereas removal of the anti-CD40 signal induces plasma cell differentiation (Arpin et al., 1995).

The CD40 culture system described by Banchereau et al. (1991), in which highly purified B cells can be maintained for long periods through cross-linking surface CD40 in the presence of IL-4, opened up new possibilities for the *in vitro* study of human B lymphocytes. However, there have been conflicting reports about the suitability of this system for antigen-driven B-cell expansion and specific antibody production *in vitro*. Nonoyama et al. (1993) report that the continued presence of antigen, anti-CD40, and IL-10 enabled the induction of phage-specific antibody whereas Callard et al. (1995) found that CD40 cross-linking in the presence of IL-2-inhibited specific (influenza virus) antibody production by purified human B cells.

We show in the present report that the "standard" CD40 system fails to sustain specific antibody-secreting B cells, an effect that might be due to overgrowth of nonspecific B-cell clones. However, preselection of specific B cells before placing in culture with CD32/L cells, anti-CD40, and IL-4 to induce clonal expansion, followed by plasma cell induction with IL-2 and IL-10, enables the detection of specific antibody. In addition, prolonged contact with antigen during stimulation with anti-CD40 and IL-4 appears to inhibit the subsequent formation of antibody-producing plasma cells.

### RESULTS

### Antibody Production in FACS-Sorted Splenic B Cells from a CMV-Positive Donor Cultured in the CD40 System

During preliminary experiments to examine the response of memory B cells in the CD40 culture system, we used B cells from individuals known to have been exposed to various antigens such as tetanus toxoid (vaccination) or cytomegalovirus (natural or iatrogenic infection). FACS-sorted splenic B cells ($CD20^{+}$; > 98% pure) were cultured with CD32/L cells, anti-CD40 (Mab89), and IL-4. At days 7 and 14, the medium was refreshed and IL10/IL-2 were added to induce plasma cell differentiation. The majority of cells showed good proliferation that was maintained for at least 4 weeks, however, we were only able to detect a very low level of CMV-specific antibody (one anti-CMV antibody-producing culture out of 144 in three separately performed experiments using the same donor material). In contrast, pokeweed mitogen (PWM) stimulation of splenic mononuclear cells (MNC) from similar donors latently infected with CMV showed IgG anti-CMV antibody in 2/3 cases (at least one positive well per 150,000 cells compared to one per 5,500,000 cells in the CD40 cultures). A similar pattern was found for the IgG anti-tetanus antibody (results not shown). These last results suggest that the frequency of specific B cells is not the limiting factor and that specific antibody may be undetectable due to preferential stimulation of nonspecific B clones or of a particular splenic B-cell compartment.

### Addition of CMV Antigen to CD40 Cultured Splenic B Cells

Firstly, we asked if the addition of CMV antigen to the cultures could provide a stimulating effect by inducing a higher frequency of detectable antigen-specific antibody. Highly purified $CD20^{+}$ splenic B cells from CMV-positive donors were placed into culture with CD32/L cells, anti-CD40, IL-4, and antigen. Antigen was in the form of (1) a cell lysate of CMV LA-infected fibroblasts, (2) the recombinant CMV protein: pp65, or (3) as immune complexes (consisting of CMV recombinant proteins, r.pp65, complexed with mouse IgG1 anti-pp65, Mab C10; or m.IgG2a anti-pp65, C12). Vigorous B-cell proliferation was observed at day 4 in all wells where anti-CD40 and IL-4 were present and the presence of antigen in any form did not increase this. See Figure 1. One exception, however, was in the case of the

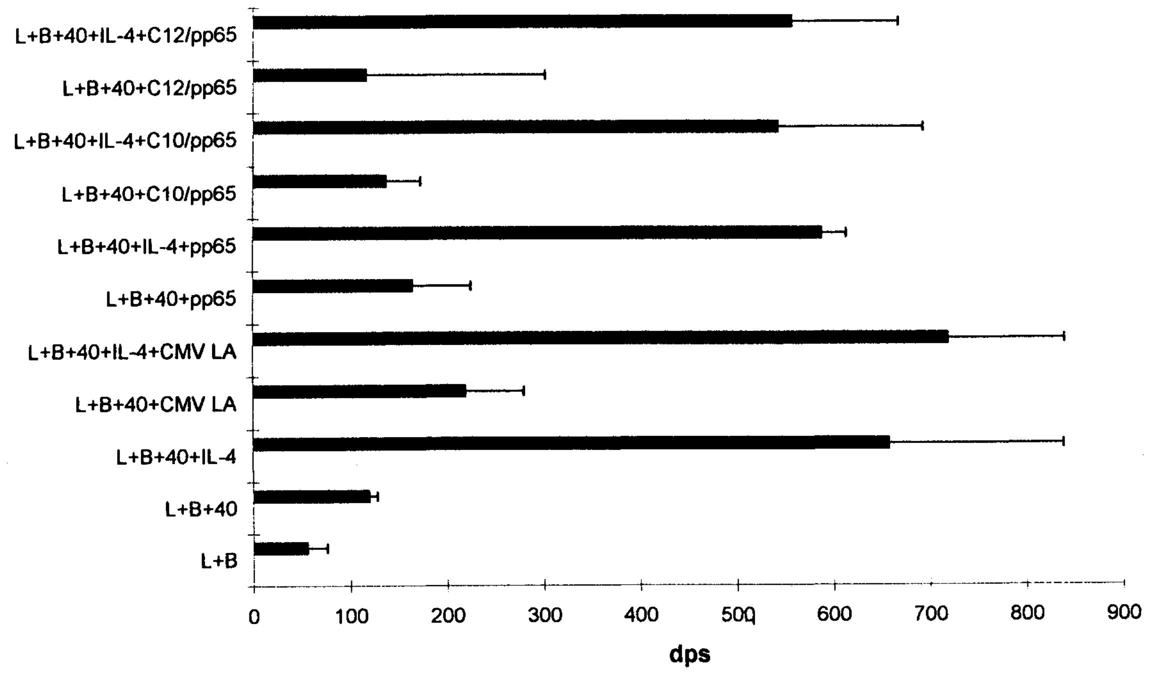

FIGURE 1 Addition of antigen in the form of soluble CMV proteins or as immune complex does not increase the proliferation at day 4 of anti-CD40/IL-4 cultured B cells. After 7 days, culture was continued by adding IL-2/IL-10 to induce plasma cell differentiation, however, no CMV-specific antibody could be detected. Antigen was added in the form of (a) cell lysate of CMV LA-infected fibroblasts, (b) recombinant CMV protein; r.pp65, or (c) immune complexes as described in Materials and Methods. Proliferation (3H-Thymidine uptake) was determined after 4 days and expressed as the mean dps ±SD of triplicate cultures. L = CD32 transfected L cells; B = B lymphocytes; IL-4 = interleukin-4; 40 = anti-CD40; CMV LA = lysate of CMV-infected fibroblasts; Mab. 89. C12 = m.IgG2a anti-pp65; C10 = m.IgG1 anti-pp65; pp65 = recombinant pp65.

CMV cell lysate (CMV LA) that appeared to have an additional mitogenic effect with a large increase in the size and number of clones per well observed microscopically at day 7 (data not shown). The data in Figure 1 illustrate the strong B-cell proliferation induced in all anti-CD40/IL-4 cultures either with or without antigen. After a further 7 days culture with IL-10/IL-2, IgM (250-350 ng/ml) and IgG (20-50 ng/ml) were detected in all wells, but CMV-specific antibody could not be detected at any time.

### Dual Triggering by Anti-CD40 and Ag to Selectively Activate the Antigen-Specific B Cells

Next we asked if we could selectively activate the antigen-specific B cells by dual triggering of CD40 and the BCR but without the additional stimulation of cytokines. B-cell cultures were set up with anti-CD40, immobilized on CD32/L cells together with antigen (in the form of soluble protein or immune complexes as before), but without IL-4 for the first 4 days. In separate experiments, we had found that CD40/Ag-stimulated B cells showed some limited proliferation for up to 4 days in the absence of IL-4 and could be "rescued" for continued growth by transfer to fresh CD40/CD32/L cultures with IL-4. After 5 days (day 9), IL-10/IL-2 were added to induce plasma cell differentiation and medium and cytokines were refreshed again at 7-day intervals.

By restricting the signals to anti-CD40 and antigen for the first 4 days, it was hoped to preferentially activate only those B cells specific for one of the CMV proteins. Total IgM and IgG in cultures where signals were restricted to anti-CD40 and CMV antigens was considerably less than in control wells (CD40/IL-4), which would be expected with a selective stimulation of specific B cells (Figure 2). However, again CMV-specific antibody could not be detected. This experiment shows that although 4 days of CD40/Ag stimulation permits survival of (a subset of) B cells without IL-4, the subsequent Ig production by the rescued B cells does not include any detectable specific antibody, suggesting that selective stimulation and expansion of Ag-specific B cells did not occur.

### Culture of Antigen-Selected B Cells in CD40 System. Panning of Peripheral Blood B Cells from Patients with an Active CMV Infection

In order to increase the starting number of antigen-specific cells, peripheral blood from donors who had recently experienced an active secondary CMV infection (high IgG anti-CMV antibody levels in serum) was chosen as source of specific B cells. In addition, a selection procedure was carried out by panning (Steenbakkers et al., 1994) CMV-specific B cells over CMV LA or recombinant pp65 (r.pp65) or r.gH. Similarly, CMV-specific B cells from a $CMV^+$ spleen were selected by panning. The selected B cells were cultured as described and supernatant was removed for analysis at 7-day intervals and replaced by fresh culture medium and the relevant cytokines. On microscopic examination, small clusters of B cells were observed in most culture wells during the first 7 days. However, the compact clusters characteristic of anti-CD40-stimulated B cells were considerably smaller than seen in nonselected B-cell cultures. After the addition of IL-10 and IL-2, increasing numbers of

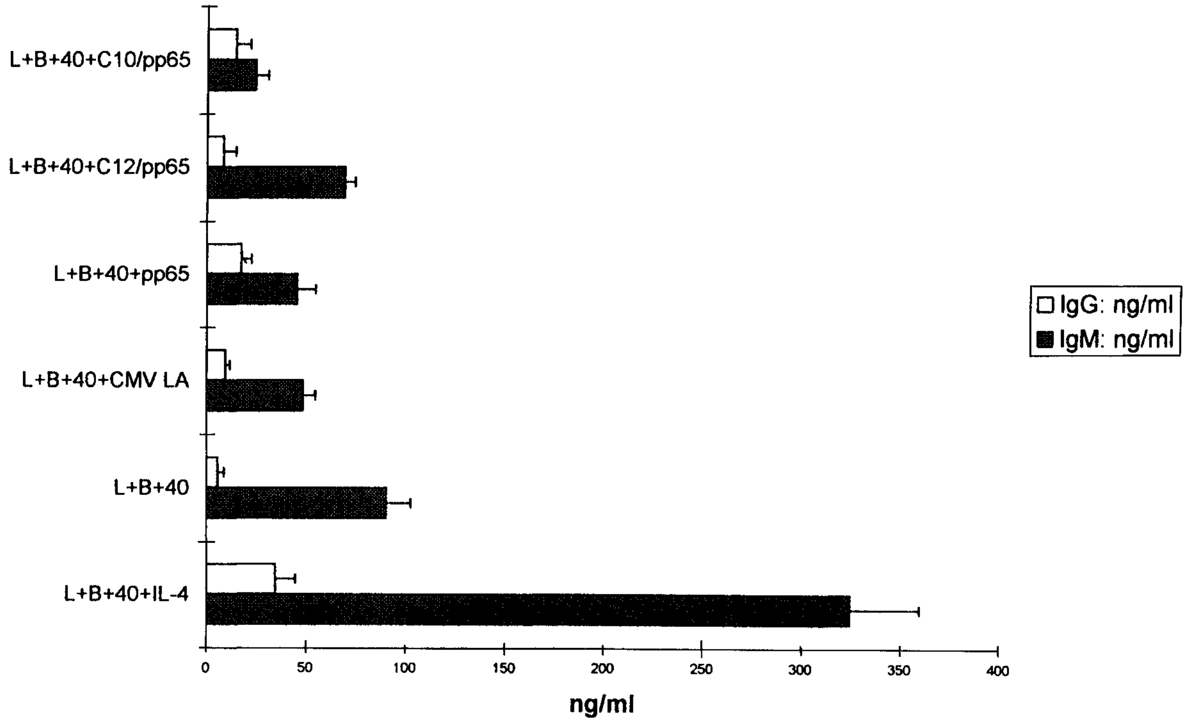

FIGURE 2 Antibody production after dual triggering of CMV-specific B cells with antigen and anti-CD40. Highly purified human B cells were stimulated 4 days with anti-CD40 and CMV antigens but without IL-4. Antigen was in the form of soluble protein or immune complexes as in Fig. 1. The surviving B cells were rescued by transfer to fresh anti-CD40 cultures containing IL-4. After 5 days, culture was continued in IL-10/IL-2. This figure shows total IgM and IgG production in the culture supernatant at day 14. Anti-CMV antibody was not detectable in any of these cultures. The data shown represent the mean ± SD of triplicate cultures. L = CD32-transfected L cells; B = B lymphocytes; IL-4 = interleukin-4; 40 = anti-CD40; CMV LA = lysate of CMV-infected fibroblasts; Mab. 89. C12 = m.IgG2a anti-pp65; C10 = m.IgG1 anti-pp65; pp65 = recombinant pp65.

TABLE I The Number of Cultures Containing Total IgM or IgG or Specific Antibody at Day 14 in CD40-Cultured Peripheral Blood or Splenic B Cells after Panning

| B cells[a] | Antigen[b] (panning) | IgG + IgM positive/wells seeded | Ag-specific Ig (no. wells) | Ag-specific/ total seeded (%) |
|---|---|---|---|---|
| 1. FACS isolated spleen | None | 48/48 | 0 | 0 |
| 1. FACS isolated spleen | CMVLA | 8/8 | 1 | 12.5 |
| 2. PB/MNC | CMVLA | 33/48 | 13 | 27.1 |
| 2. PB/MNC | CMV r.pp65 | 16/40 | 1 | 2.5 |
| 2. PB/MNC | CMV r.gH | 5/64 | 2 | 3.1 |
| 2. PB/MNC | TT | 5/6 | 3 | 50 |

[a]CD20$^+$ sorted splenic B cells were panned or placed directly in culture as described. Peripheral blood mononuclear cells (PB/MNC) were panned over antigen as indicated.
[b]Antigen: CMV LA = lysate of CMV-infected fibroblasts; CMV r.pp65 = recombinant CMV glycoprotein; and CMV r.gH = recombinant CMV glycoprotein.

free lying plasma cells were observed, as is typical in the nonselected B-cell cultures.

Optimal Ig production was found in the culture supernatants harvested between days 14 and 17 with CMV-specific antibody only being detectable from day 14 onwards. This suggests that the specific antibody producers have first undergone a proliferative phase under influence of anti-CD40 and IL-4 before differentiating to antibody-secreting B cells in the presence of IL-10/IL-2. That nearly all wells show good Ig production proves the presence of functional B cells (Table I).

The data in Table 1 show that CMV-specific B cells can be selected by panning and that the frequency of specific-antibody-producing wells depends on the nature of the antigen used for selection. The panning experiments with the highest number of wells containing antibody specific for CMV were those where CMV LA was used to select B cells, which is to be expected since the CMV LA lysate contains a wide variety of CMV antigens (see Table 1). In a comparable experiment, tetanus-specific B cells were selected by panning over TT and cultured as described for CMV-specific B cells. Here, antigen-specific clones were obtained in 50% of the culture wells. (Table 1). These results suggest a higher precursor frequency for tetanus-toxoid-specific B cells than for CMV.

It appears from this work that specific B cells selected by panning and placed in a two-phase culture system differentiate to specific-antibody-producing plasma cells. However, it was not possible to say if the initial CD40/IL-4 phase actually induced expansion of the selected B cells because too few cells were available for a proliferation assay.

### Prolonged Contact with Antigen Inhibits Specific Antibody Production in CD40-Stimulated B Cells

The low frequency of antibody-producing cultures leads us to ask whether the duration of contact with antigen during or after panning might influence B-cell responsiveness.

In order to test this, peripheral blood MNC from two donors recently boosted for tetanus were placed in tetanus-toxoid-coated plates and the specific B cells were allowed to adhere following the procedure for panning as described above. After removal of the nonadherent cells by thorough washing, the irradiated CD32/L cells, anti-CD40, and IL-4 were added to the panning plates and allowed to incubate either overnight or for 4 days. At the end of the initial 24-hour or 4-day culture period, the cells were removed, washed, and transferred to freshly irradiated CD32/L cells with anti-CD40 and IL-4 for a further 5 days. In each case, the medium was refreshed and the cultures were continued with IL-10/IL-2. Supernatant was harvested at day 14, 17, and 21. Antigen-specific antibody was detected when the panned B cells were washed to remove antigen and transferred to fresh cultures after 1 day, but could not be detected in either donor when the B-cell cultures were continued in the panning

TABLE II Total IgM, IgG, and Tetanus-Specific B-Cell cultures Following 1 or 4 Days Incubation with Antigen

| | | No. positive wells at day 17[a] Transfer day 1 | Transfer day 4 |
|---|---|---|---|
| Donor 1 | IgM | 3 | 4 |
| | IgG | 4 | 4 |
| | TT[b] | 2 | 0 |
| Donor 2 | IgM | 6 | 6 |
| | IgG | 6 | 6 |
| | TT | 3 | 0 |

[a]Tetanus-specific B cells were selected and cultured in the presence of antigen for 1 day or 4 days with CD32/L cells, anti-CD40, and IL-4 before washing and transferring to 24-well plates with anti-CD40/IL-4. After a further 5 days, the medium was refreshed and culture continued with IL-2/IL-10.
[b]TT = tetanus toxoid.

plates for 4 days before removal of antigen (Table II). This suggests that the continued presence of antigen during culture inhibits the differentiation to plasma cells in the specific B-cell population only since the total IgM and IgG secretion was not affected. Also the effect appears to be long lasting and could not be reversed by the subsequent removal of antigen and further culture with IL-10/IL-2.

### DISCUSSION

The most important finding from this study is that prior selection of CMV-specific B cells enables the detection of specific antibody in CD40-cultured B cells that was not readily detectable in nonselected polyclonal B cell cultures. We conclude that the antigen-specific (CMV memory) B cells respond to anti-CD40/cytokine-induced differentiation. An important question that remains to be answered is whether the lack of detection of specific antibody in nonselected cultures is due to the extremely low frequency of B memory cells or whether the antigen-selection step (panning) gave an initial activation signal that enabled subsequent response in the CD40 cultures. Furthermore, specific antibody was detected generally only after 14 days using the present protocol, suggesting, as indicated by others (Arpin et al., 1995; Callard et al., 1995; Silvy et al., 1996), that the continuing presence of anti-CD40 may delay antibody production. Secondly, we found that the continued presence of antigen, at least for the first 4 days of anti-CD40 culture, appeared to abolish the specific antibody response from antigen-selected B cells. This effect was long lasting and specific antibody production could not be restored by subsequent removal of antigen and continued culture with IL-10/IL-2 up to 21 days.

In the present study, our aim was to utilize the proliferative properties of the combination anti-CD40/IL-4 to obtain (clonal) expansion of the selected B memory cells and then extended the culture period to allow differentiation to antibody-producing cells under the influence of IL-2/IL-10. Several reports show that CD40 stimulation of B memory cells ($IgD^-/CD38^-$) *in vitro* leads to proliferation rather than differentiation to plasma cells (Arpin et al., 1995; Callard et al., 1995; Silvy et al., 1996) and that the differentiation of memory B cells into antibody-secreting cells can be achieved on costimulation with IL-2 and IL-10 without the engagement of CD40. In the present study, we expect a decreased effect of anti-CD40 after 14 days culture and this may contribute to the increased detection of antigen-specific antibody measured after this time.

A notable feature of these cultures was that the degree of proliferation observed in peripheral blood (antigen-selected) B cells appeared limited compared to what we expected on the basis of comparable cultures of (antigen-selected or -unselected) spleen B cells. This reflects an important difference between small circulating B cells that generally have a low expression of the B-cell "preactivation" marker, CD21, and the spleen B-cell population containing anywhere from 5-50% marginal-zone B cells that are $CD21^{high}$. Although blood and spleen (comprising centrocytes, centroblasts, marginal zone, mantle zone, and PB B cells) represent quite different B-cell compartments, the results illustrate that enrichment for antigen-specific B cells from both populations is possible using this technique. In these preliminary experiments, no attempt was made to characterize the ability of the different B-cell subsets to respond in the CD40 cultures.

The effect of different doses of antigen was examined in the CD40/IL-4 cultures of the non-selected B cells, but no effect on specific antibody production was detected. It is likely that the effect of varying antigen concentration is masked by the continuing presence of anti-CD40 that inhibits antibody production. No attempt was made to vary antigen concentration with B cells selected by panning since a certain degree of antigen coating on plastic panning trays is inherent in the panning technique. It should also be remembered that it is not only the antigen concentration that may affect B cell activation, but also the time of exposure to antigen, as shown by the present results.

CD40 and IL-4 costimulation has been reported to induce proliferation and prevent the apoptosis mediated by hyper cross-linking of $IgM^+/IgD^+$ peripheral B cells (Parry et al., 1994). A proliferation assay of the antigen-specific B cells in the current experiments was not performed due to the extremely low numbers of specific B cells harvested after panning. In an alternative system for expanding antigen-specific B cells using an EL-4 thymoma line and T-cell culture medium (Steenbakkers, 1994; Lagerkvist et al., 1995), proliferation of the selected B cells is clearly visible up to about day 10. However, this system results in B cells lying free (personal observation) as opposed to tight aggregates as observed in the CD40 culture system. Although EL-4 cells also ligate CD40 on B cells, the mixture of different cytokines in the T-cell supernatant may explain the difference in observed proliferation. The T-cell replacing factor used with the EL-4 cultures is either a PWM-stimulated T-cell supernatant (Lagerkvist et al., 1995) or a PHA-stimulated T-cell-macrophage supernatant (Steenbakkers, 1994), and the cytokines thus induced are likely to resemble those present in the PWM-stimulated MNC cultures yielding the anti-CMV antibody described at the beginning of this report. Also the cytokine and phorbol myristate acetate (PMA) induced induction of gp39 on the EL-4 cells may more closely resemble transient gp39 expressed on T cells than the long-term triggering via the CD32-bound monoclonal antibody in the CD40 system.

The second important finding in the present study is that the presence of antigen during the first 4 days of culture appeared to abolish the specific antibody response from antigen-selected B cells. It is possible that BCR ligation by antigen during panning for specific B cells provides an important costimulation together with anti-CD40, but that the continued presence of antigen during stimulation with anti-CD40 may render these cells unresponsive. A similar phenomenon has been described *in vivo* where soluble antigen administered during the germinal center reaction can cause enhanced apoptosis of germinal center B cells and reduce B memory cell formation (Pulendran et al., 1995; Shokat and Goodnow, 1995). Supporting this, Galibert et al. (1996b) have reported that prolinged BCR cross-linking of CD40-activated GC B cells can lead to negative selection of these cells. Further experiments are necessary to determine if the abolition of the specific antibody response reported here is dependent on the concentration and valency of the antigen. In the present study, the antigen concentration was determined by the amount of antigen bound to the panning plates after incubation of the antigen coating solution overnight.

It is interesting to compare the present findings with those of Callard et al. (1995), who describe good proliferation but inhibition of the specific antibody (influenza) response after CD40 cross-linking of highly purified tonsil B cells. There are a number of important differences in their approach such as the absence of CD32/L cells for cross-linking anti-CD40 and the use of a different anti-CD40 Mab. Most significant, however, is the presence of antigen for the first 6 days of culture with anti-CD40 and culture in IL-2 instead of IL-2/IL-10, as described in the present experiments. They conclude that it is the CD40 crosslinking that inhibits specific antibody production by human B cells, but did not consider the fact that it may be the long-term dual triggering of the BCR and anti-CD40 that is having this effect. In addition, whereas T cells have been found to activate B cells in a CD40- and IL-2-dependent fashion (Blanchard et al., 1994) and IL-2 was essential for the anti-influenza antibody production by immunized MNC *in vitro* (Callard et al., 1995), IL-2 alone appears unable to activate B cells in the CD40 system (Blanchard et al., 1994). This is in contrast to BCR stimulation by antigen or SAC in the absence of anti-CD40. We and others have found IL-2 to augment the specific (Callard et al., 1995) or polyclonal antibody production in BCR-triggered B cells *in vitro* (Jelenek and Lipsky, 1988; Splawski et al., 1993; Itoh et al., 1994, Schilizzi et al., 1997).

The inhibition of specific antibody production by the continued presence of antigen may explain our inability to selectively trigger antigen-specific B cells by simultaneous stimulation with anti-CD40 and CMV antigens in the absence of cytokines, because in these experments, antigen was also continuously present. Wheeler et al. (1993) have found that engagement of CD40 lowers the threshold for activation of resting B cells via the antigen receptor when the antigen receptor is triggered with IgG1 anti-$\mu$ bound by its Fc region to CD32/L cells. However, they did not report the effect of coligation of CD40 and BCR on antibody production in these experiments.

In contrast to our results, Nonoyama et al. (1993) have reported antigen-specific antibody produced *in vitro* by CD40-triggered human B cells cultured for 12 days in the presence of antigen and IL-10. They selected $CD19^+/IgD^-$ peripheral blood B cells from volunteers recently immunized and boosted with the phage $\phi$ X174 and found that *in vitro* antigen stimulation was essential and synergistic with anti-CD40 and IL-10. The presumably high frequency of the specific B cells in their system may explain this discrepancy since in their experiments it was not necessary to select the phage-specific B cells before culture. Other factors determining B-cell responsiveness and that make it difficult to compare with our antigen-stimulated cultures are the nature and concentration of the antigen (Bachmann et al., 1993). Also, direct comparison is not possible since we did not perform any cultures where specific B cells or total B cells were stimulated with anti-CD40, antigen, and IL-10 as the only cytokine present.

It is not known whether naive cells preferentially survive in culture following CD40 ligation and Il-4 or whether it is a question of overgrowth of nonspecific B cells that hinders detection of a specific antibody. Furthermore, a complicating factor is that CD40 ligation initially stimulates proliferation and reduces antibody production (Arpin et al., 1995, Schilizzi et al., manuscript in preparation). So the question is really whether naive B cells preferentially survive in this system, whether they overgrow the memory B cells, or whether the antibody production of the memory B cells is being suppressed due to the continuing presence of anti-CD40.

This study supports a model for B-cell activation by T-dependent antigens in which CD40 ligand and cytokines costimulate with BCR ligation and that these signals are likely to be sequential and transient in nature. *In vivo*, CD40-CD40 ligand contact is short-lasting and tightly regulated by mechanisms such as internalization of CD40 ligand in T cells (Yellin et al., 1994). Multiple-step culture experiments are needed to unravel the key factors in the induction and differentiation of B memory cells.

### MATERIALS AND METHODS

### Reagents

Purified rIL-4 and rIL-10 were obtained as gifts from Schering-Plough Research (Kenilworth, NJ) and used at 100 U/ml and 100 ng/ml, respectively. Purified r.IL-2 (Cetus, Amsterdam) was used at 10 ng/ml and anti-CD40 (Clone 89; Immunotech, France) was used at a final concentration of 0.5 $\mu$g/ml. Mouse anti-human CD20-FITC and mouse IgG1 anti-pp65 were kindly provided by MCA Development b.v. (Groningen, The Netherlands).

**Cell Lines**

Mouse Ltk$^-$ cells transfected with the gene for human CD32 (CD32-L cells) were obtained from H. Kluin-Nellemans (Department of Hematology, AZ Leiden), with permission from J. Banchereau (Schering-Plough, Dardilly, France). The cells were maintained in the RPMI 1640 culture medium described below, to which was added HT supplement (GIBCO 50x) and 0.1 mM aminopterin (GIBCO, Paisley, Scotland).

**B-Cell Preparation**

The B cells used in the present experiments were (a) CD20$^+$ FACS-purified splenic B cells from one subject, used as a total population or after panning the CD20-positive population over antigen-coated plastic, or (b) peripheral blood cells cultured either as unfractionated mononuclear cell suspensions or as B cells enriched by panning over antigen-coated plastic plates. Spleen was obtained immediately postmortem from individuals with no known illness or current infection. Both spleen and PB cells were from CMV-positive subjects. The different B-cell populations studied are shown in Table 1.

**Spleen Cells**

Mononuclear cells were isolated from spleen, by density-gradient centrifugation over Lymphoprep (Nycomed, Oslo) and subsequently stored at 20 × 10$^6$/ml, at −80°C. Aliquots of splenic MNC were thawed as required, and any dead cells were removed by centrifugation over Lymphoprep, washed twice in RPMI 1640 medium (GIBCO), and labeled with anti-CD20 FITC. B cells were obtained by FACS-STAR (Becton-Dickinson, Mountainview, CA) single-cell sorting to yield B-cell suspensions of greater than 98% purity. The cells were washed once and resuspended at 4 × 10$^5$/ml in a culture medium consisting of RPMI 1640 supplemented with 60 $\mu$g/ml gentamicine, 1 mM Na pyruvate, 2 mM glutamine, 0.05 mM $\beta$-ME, and 15% FCS (BioWhittaker, Verviers, France). These cells were either placed directly in CD40 cultures (see "B-Cell Cultures," below) or used for panning similarly to monocyte-depleted blood-cell suspensions (see "Panning," below).

**Peripheral Blood**

MNC from two tubes of heparinized peripheral blood from patients with an active CMV infection were isolated over lymphoprep. The cells were then washed, resuspended to about 2 × 10$^6$/ml in culture medium, and adherent cells were removed by incubating for 30 min at 37°C in a 75-cm$^3$ culture flask placed horizontally. The nonadherent, MNC fraction was used for panning over plastic-coated antigen to select specific B cells or in MNC cultures, as described in below.

**Panning Specific B Cells for the CD40 System**

The panning procedure was performed exactly as described by Steenbakkers et al. (1994). The monocyte-depleted PB-cell suspensions or CD20$^+$ purified spleen B cells were then added to six-well culture plates (Costar, Cambridge, MA) that had been coated with antigen overnight at 4°C. The antigen used was either an extract of CMV LA (CMV late antgen)-infected fetal lung fibroblasts or the recombinant proteins pp65 and gH. The optimal concentration for antigen coating was tested by ELISA using a human mAb specific for pp65 (07b) or gH-reactive patient serum. The detection of CMV LA, r.pp65, and gH in this way showed that both the CMV-infected fibroblast extract and recombinant proteins used for panning are in a form able to be recognized by B cells bearing sIg specific for CMV.

After incubating the MNC or purified B cells in the panning plates for 90 min at 37°C and 5% $CO_2$, the unbound cells were removed and the wells were washed six times with PBS. The bound B cells were removed by trypsinization that was stopped with the addition to each plate of a 5-ml culture medium containing 10% FCS. The harvested cells were placed in culture without further washing. The selected B cells were resuspended in a 5-ml culture medium and divided over 48 wells of a microtiter plate in which irradiated CD32-L cells (2 × 10$^4$/well) had been placed. Anti-CD40 and cytokines were added as described in what follows (see "CD40 System").

### B-Cell Cultures: CD40 System

(a) For measurement of specific and total Ig production, the FACS-sorted human splenic B cells were seeded in triplicate or quadruplicate in 96-well culture plates (Costar, Cambridge, MA.) at $4 \times 10^4$/well on irradiated (80 GY) CD32-L cells ($2 \times 10^4$/well). Anti-CD40 (mAb89) and the cytokines IL-4 or IL-10 and IL-2 were added as indicated in a final volume of 200 $\mu$l per well. Antigen-selected (panned) B cells were not counted but divided over 48 wells of the 96-well culture plate and cultured as described above. Culture medium was refreshed after 7 days by replacing 100 $\mu$l of the medium and the addition of fresh cytokines. The supernatant was generally harvested after 7 and 14 days for quantitation of secreted Ig. In some experments, supernatant was also collected at days 17 and 21.

(b) For proliferation assays, CD32/L cells were irradiated and seeded into 96-well round-bottom tissue-culture plates (Costar). Human splenic B cells ($10^5$/well) and various combinations of cytokines were added as indicated in the following section. [$^3$H]-thymidine (sp.act. 2 Ci/mmol; Wilmington, DE) was added for the last 16 hr (1 $\mu$Ci/well) of a 4-day culture and incorporation was measured by liquid scintillation counting.

### MNC Cell Cultures: CD40 or PWM Controls

### Specific and Total Ig Production by Peripheral Blood MNC Cultures

MNC from healthy volunteers or patients with an active CMV infection were isolated by density-gradient centrifugation over Lymphoprep (Nycomed). After washing twice in RPMI 1640, the MNC were resuspended in RPMI culture medium and seeded at $2 \times 10^5$/well in 96-well round-bottom plates (Costar) in the presence of PWM or anti-CD40 and CD32-L cells.

### Analysis of Antibody Responses

Culture supernatants were analyzed by ELISA for the presence of total human IgM and IgG as well as antibodies specific for the CMV antigens pp65, gH, and CMV LA. In some experiments, anti-tetanus antibodies were measured. The antigens used for coating were (a) CMV LA (late antigens) prepared as described (Middeldorp et al., 1984), (b) r.pp65 prepared as described by van Zanten et al. (1995), (c) r.gH prepared similarly, or (d) tetanus toxoid (SVM, Bilthoven, The Netherlands). The CMV-specific ELISA was performed as described previously (van der Giessen et al., 1990) with some slight modifications. Briefly, microtiter plates (Dynatech, Alexandria, VA) were coated 48 hr with 1 $\mu$g/ml of antigen (CMV proteins or tetanus toxoid) or goat anti-human IgM or IgG (Biosource, Camarillom, CA) in 50 mM carbonate buffer, pH 9.6. Alkaline phosphatase conjugated goat anti-human IgM and goat anti-human IgG (SBA, Birmingham, AL) were used separately or combined as required, to detect bound antibodies. The substrate used was Nitrophenyl phosphate disodium (Sigma Chemical, St. Louis), dissolved in diethanolamine buffer 10%, pH 9.8. The CMV-specific monoclonal antibody (mab 07b) or CMV-positive patient serum was used as a positive control in the CMV-specific ELISA. The concentrations of IgM and IgG in the supernatant were determined by comparison with a standard curve using a standard human serum (Behringwerke AG, Marburg, Germany).

### *References*

Armitage R.A., Macduff B., Spriggs M.K. and Fanslow W. (1993). Human B cell proliferation and Ig secretion induced by recombinant CD40 ligand are modulated by soluble cytokines. J. Immunol. **150**: 3671-3680.

Arpin C., Dechanet J., van Kooten C., Merville P., Grouard G., Briere F., Banchereau J. and Liu Y.L. (1995). Generation of memory B cells and plasma cells in vitro. Science **268**: 720-722.

Bachmann M.F., Rohrer U.H., Kundig T.M., Burki K., Hengartner H. and Zinkernagel R.M. (1993). The influence of antigen organisation on B cell responsiveness. Science **262**: 1448-1451.

Banchereau J., de Paoli P., Valle A., Garcia E. and Rousset F. (1991). Long-term human B cell lines dependent on interleukin-4 and antibody to CD40. Science **251**: 70-72.

Blanchard D., Gaillard C., Hermann P. and Banchereau J. (1994). Role of CD40 and interleukin-2 in T cell-dependent human B lymphocyte growth. Eur. J. Immunol. **24**: 330-335.

Callard R.E., Herbert J., Smith S.H., Armitage R.J. and Costelloe K.E. (1995). CD40 cross-linking inhibits specific antibody production by human B cells. Int. Immunol. **7**: 1809-1815.

Clark E.A., Yip T.C., Ledbetter J.A., Yukawa J., Kikutani H., Kishimoto T. and Ng M.H. (1988). Cdw40 and BLCa-specific monclonal antibodies detect two distinct molecules which transmit progression signals to human B lymphocytes. Eur. J. Immunol. **18**: 451-457.

Galibert L., Burdin N., Barthelemy C., Meffre G., Durand I., Garcia E., Garrone P., Rousset F., Banchereau J. and Liu Y-J. (1996b). Negative selection of human germinal center B cells by prolinged BCR cross-linking. J. Exp. Med. **183**: 2075-2085.

Galibert L., Burdin N., de Saint-Vis B., Garrone P., van Kooten C., Banchereau J. and Rousset F. (1996a). CD40 and B cell antigen receptor dual triggering of resting B lymphocytes turns on a partial germinal center phenotype. J. Exp. Med. **183**: 77-85.

Gascan H., Gauchat J.F., Aversa G., van Vlasselaer P. and De Vries J.E. (1991). Anti-CD40 monoclonal antibodies or CD4+ T cell clones and IL-4 induce IgG4 and IgE switching in purified human B cells via different signalling pathways. J. Immunol. **147**: 8-13.

Hollenbaugh D., Grosmaire L.S., Kullas C.K., Chalupny N.J., Braesch-Andersen S., Noelle R.J., Stamenkovic J.A., Ledbetter J.A. and Aruffo A. (1992). The human T antigen gp39, a member of the TNF gene family, is a ligand for the CD40 receptor: Expression of a soluble form of gp39 with B cell co-stimulatory activity. EMBO J. **11**: 4313-4321.

Itoh K., Inoue T., Ito K. and Hirohata S., (1994). The interplay of interleukin-10 (IL-10) and interleukin-2 (IL-2) in humoral immune responses: IL-10 synergises with IL-2 to enhance responses of human B lymphocytes in a mechanism which is different from upregulation of CD25 expression. Cell. Immunol. **157**: 478-488.

Jelenek D.F. and Lipsky P.E. (1988). Inhibitory influence of IL-4 on human B cell responsiveness. J. Immunol. **141**: 164-173.

Lagerkvist A.C., Furebring C. and Borrebaeck C.A. (1995). Single, antigen-specific B cells used to generate Fab fragments using CD40-mediated amplification or direct PCR cloning. Biotechniques **18**: 862-869.

Liu Y., Johnson G.D., Gordon J. and MacLennan I.C.M. (1992). Germinal centres in T-cell-dependent antibody responses. Immunol. Today **13**: 17-21.

Middeldorp J., Jongsma J., ter Haar A., Schirm J. and The T.H. (1984). Detection of immunoglobulin M and G antibodies against cytomegalovirus early and late antigens by enzyme-linked immunosorbent assay. J. Clin. Microbiol. **20**: 763-771.

Nonoyama S., Hollenbaugh D., Aruffo A., Ledbetter J.A. and Ochs H.D. (1993). B cell activation via CD40 is required for specific antibody production by antigen-stimulated human B cells. J. Exp. Med. **178**: 1097-1102.

Parry S.L., Hasbold J., Holman M. and Klaus G.G.B. (1994). Hypercross-linking surface IgM or IgD receptors on mature B cells induces apoptosis that is reversed by co-stimulation with IL-4 and anti-CD40. J. Immunol. **152**: 2821-2829.

Puledran B., Kannourakis G., Nouri S., Smith K.G.C. and Nossal G.J.V. (1995). Soluble antigen can cause enhanced apoptosis of germinal-centre B cells. Nature **375**: 331-334.

Rousset F., Garcia E., Defrance T., Peronne C., Vezzio B., Hsu D., Kastelein R., Moore K.W. and Banchereau J. (1992). Interleukin 10 is a potent growth and differentiation factor for activatd human B lymphocytes. Proc. Natl. Acad. Sci. USA **89**: 1890-1893.

Rousset F., Peyrol S., Garcia E., Vezzzio N., Andujar M., Grimmaud J.-A. and Banchereau J. (1995). Long-term cultured CD40-activated B lymphocytes differentiate into plasma cells in response to IL-10 but not IL-4. Int. Immunol. **7**: 1243-1253.

Schilizzi B.M., Boonsta R., The T.H., and De Leij L.F.M.H. (1997) Effect of B-Cell receptor engagement on CD40-stimulated B cells. Immunology **92**: 346–353.

Shokat K.M. and Goodnow C.C. (1995). Antigen-induced B-cell death and elimination during germinal-centre immune responses. Nature **375**: 334-338.

Silvy A., Lagresle C., Bella C. and Defrance T. (1996). The differentiation of human memory B cells into specific antibody-secreting cells is CD40 independent. Eur. J. Immunol. **26**: 517-524.

Splawski J.B., Shu Man Fu and Lipsky P.E. (1993). Immunoregulatory role of CD40 in human B cell differentiation. J. Immunol. **150**: 1276-1285.

Steenbakkers P.G.A, Hubers H.A.J.M. and Rijnders W.M. (1994). Efficient generation of monoclonal antibodies from preselected antigen-specific B cells. Mol. Biol. Reports **19**: 125-134.

van der Giessen M., van den Berg A.P., van der Bij W., Postma S., van Son W.J. and The T.H. (1990). Quantitative measurement of CMV-specific IgG and IgM antibodies in relation to CMV antigenemia and disease activity in kidney recipients with an active CMV infection. Clin. Exp. Immunol. **80**: 56-61.

Van Zanten J., Harmsen M.C., van der Giessen M., et al. (1995). The humoral immune response against human cytomegalovirus (HCMV)-specific proteins after HCMV infection in lung transplantation. Significance of epitopes present on prokaryotically expresssed recombinant proteins versus epitopes on naturally occurring proteins. Clin. Diag. Lab. Immunol. **2**: 214-218.

Wheeler K., Pound J.D., Gordon J. and Jefferis R. (1993). Engagement of CD40 lowers the threshold for activation of resting B cells via antigen receptor. Eur. J. Immunol. **23**: 1165-1168.

Yellin M.J., Sippel K., Inghirami G., Covey L.R., Lee J.J., Sinning J., Clark E.A., Chess L. and Lederman S. (1994). CD40 molecules induce down-modulation and endocytosis of T cell surface T cell-B cell activating molecule/CD40-L. Potential role in regulating helper effector function. J. Immunol. **152**: 598-608.

